# Drug Disposition and Pharmacotherapy in Neonatal ECMO: From Fragmented Data to Integrated Knowledge

**DOI:** 10.3389/fped.2019.00360

**Published:** 2019-09-03

**Authors:** Genny Raffaeli, Pavla Pokorna, Karel Allegaert, Fabio Mosca, Giacomo Cavallaro, Enno D. Wildschut, Dick Tibboel

**Affiliations:** ^1^Fondazione IRCCS Ca' Granda Ospedale Maggiore Policlinico, NICU, Milan, Italy; ^2^Department of Clinical Sciences and Community Health, Università degli Studi di Milano, Milan, Italy; ^3^Department of Pediatrics—ICU, General University Hospital, 1st Faculty of Medicine Charles University, Prague, Czechia; ^4^Department of Pharmacology, General University Hospital, 1st Faculty of Medicine Charles University, Prague, Czechia; ^5^Intensive Care and Department of Pediatric Surgery, Erasmus MC-Sophia Children's Hospital, Rotterdam, Netherlands; ^6^Division of Neonatology, Department of Pediatrics, Erasmus MC Sophia Children's Hospital, University Medical Center Rotterdam, Rotterdam, Netherlands; ^7^Department of Development and Regeneration, KU Leuven, Leuven, Belgium

**Keywords:** ECMO, pharmacokinetics, pharmacodynamics, critical illness, developmental pharmacology, neonate

## Abstract

Extracorporeal membrane oxygenation (ECMO) is a lifesaving support technology for potentially reversible neonatal cardiac and/or respiratory failure. As the survival and the overall outcome of patients rely on the treatment and reversal of the underlying disease, effective and preferentially evidence-based pharmacotherapy is crucial to target recovery. Currently limited data exist to support the clinicians in their every-day intensive care prescribing practice with the contemporary ECMO technology. Indeed, drug dosing to optimize pharmacotherapy during neonatal ECMO is a major challenge. The impact of the maturational changes of the organ function on both pharmacokinetics (PK) and pharmacodynamics (PD) has been widely established over the last decades. Next to the developmental pharmacology, additional non-maturational factors have been recognized as key-determinants of PK/PD variability. The dynamically changing state of critical illness during the ECMO course impairs the achievement of optimal drug exposure, as a result of single or multi-organ failure, capillary leak, altered protein binding, and sometimes a hyperdynamic state, with a variable effect on both the volume of distribution (Vd) and the clearance (Cl) of drugs. Extracorporeal membrane oxygenation introduces further PK/PD perturbation due to drug sequestration and hemodilution, thus increasing the Vd and clearance (sequestration). Drug disposition depends on the characteristics of the compounds (hydrophilic vs. lipophilic, protein binding), patients (age, comorbidities, surgery, co-medications, genetic variations), and circuits (roller vs. centrifugal-based systems; silicone vs. hollow-fiber oxygenators; renal replacement therapy). Based on the potential combination of the above-mentioned drug PK/PD determinants, an integrated approach in clinical drug prescription is pivotal to limit the risks of over- and under-dosing. The understanding of the dose-exposure-response relationship in critically-ill neonates on ECMO will enable the optimization of dosing strategies to ensure safety and efficacy for the individual patient. Next to *in vitro* and clinical PK data collection, physiologically-based pharmacokinetic modeling (PBPK) are emerging as alternative approaches to provide bedside dosing guidance. This article provides an overview of the available evidence in the field of neonatal pharmacology during ECMO. We will identify the main determinants of altered PK and PD, elaborate on evidence-based recommendations on pharmacotherapy and highlight areas for further research.

## Background

Extracorporeal membrane oxygenation (ECMO) is an established life-saving support technique for critically-ill neonates with severe cardio-respiratory failure (1, 2). Being a bridge, ECMO buys the time for cure, in part related to drugs to treat and possibly reverse the underlying disease while protecting the failing respiratory/circulatory systems from iatrogenic negative effects with long term consequences (2). Indeed, sustained and maximal mechanical ventilation may lead to hemodynamic compromise and ventilation-induced lung injury, as a result of oxygen toxicity, baro- bio-, and volu-trauma (3). Generally, these critically-ill neonates are exposed to polypharmacy, as they require anticoagulants to maintain the hemostatic balance within the ECMO circuit, analgo-sedatives to ensure patient comfort, cardiovascular agents to sustain hemodynamics, anti-infectives to prevent or treat infections, and possibly other drugs to manage underlying specific conditions or complications (4, 5).

As in many of these patients the survival and overall outcome rely on medications, effective pharmacotherapy is essential to improve care and minimize side effects (5). Adequate drug dosing is based on the understanding of two concepts: (1) pharmacokinetics (PK), which explores “what the body does to the drug” and provides the drug concentration-time profile, through the evaluation of absorption, distribution, metabolism, and excretion (ADME); (2) pharmacodynamics, which represents “what the drug does to the body” and estimates action and side-effects of a given medication, based on dose and patient profile (6, 7). The main drivers of drug PK are volume of distribution (Vd), which describes the dose required to produce the desired peak concentration and clearance (Cl), which is the volume of fluid cleared of drug from the body per unit of time. Both Vd and Cl are primary determinants of drug half-life (7). Safe and efficient prescription in neonatal ECMO depends upon the knowledge of the above-mentioned concepts and the understanding of the determinants affecting drug PK and PD in the complex context of patient immaturity, critical illness, (multi)organ failure and need for supportive extracorporeal circuits (8).

Neonatal age is by itself a window of pharmacological vulnerability (9). Drug PK and PD prediction, based on time-dependent maturational changes (age, weight) is the cornerstone of developmental pharmacology (10, 11). Additionally, critical illness may contribute to impaired drug exposure, as a result of multiple organ failure and changes in physiology, such as hyperdynamic state, increased vascular permeability, catabolism, and altered protein binding (8, 12). The need for ECMO further complicates the issue, through the sequestration of drugs into the circuit and the induction of PK specific variability (4, 13, 14).

Although physicochemical properties can be used to predict the drugs' bioavailability while on ECMO (15), the pharmacotherapy in this setting remains too empirical, as a result of limited evidence due to the lack of clinical studies and ever-evolving technology.

Because of this, treating a critically-ill neonate on ECMO is challenging and requires an integrated approach, to limit the risks of under treatment or toxicity. In this review, we will discuss current knowledge of ECMO-induced PK perturbations, and subsequently discuss the relevance of these PK findings for analgo-sedatives and antimicrobial and antiviral drugs, to end with a discussion on approaches to further optimize neonatal pharmacotherapy. However, pharmacotherapy for neonates on ECMO still needs to be integrated with the physiological maturation occurring in early infancy.

## The Role of Developmental Pharmacology on Drug Disposition

In neonates the evolving physiological maturation has a dynamic impact on clinical pharmacology, thus resulting in inter- and intra- individual variability in drug exposure (PK) and drug effect (PD) (9). Growth, weight, body and plasma protein composition, organ maturation, and energy requirements are the main determinants of the developmental pharmacology, which integrates the knowledge of the ontogenetic changes to deliver safe and effective pharmacological treatment across the pediatric age range (10, 16). While maturational PK considers the age-related changes of the ADME process (17, 18), the maturational PD takes into account the developmental variability of specific organ function and receptor expression (11). An extensive and contemporary description of the maturational covariates of the developmental pharmacology is beyond the scope of this review and it is available elsewhere (10, 11, 19).

## The Role of Non-maturational Determinants on Drug Disposition: Focus on Pre-ECMO Disease State

To objectivate non-maturational determinants and their impact on drug disposition in critically ill neonates is essential to integrate the concept of “precision dosing to optimize neonatal pharmacotherapy” defined as “personalized, individualized, tailored or precise pharmacotherapy” (20). Moreover, accuracy of drug formulations, drug prescription and new drug development is needed to tune pharmacotherapy in the vulnerable neonatal population (21). Non-maturational determinants such as (perinatal) asphyxia/hypoxia, sepsis/systemic inflammatory response syndrome (SIRS), multiple organ dysfunction syndrome (MODS) are considered as clinically relevant variables of drug disposition (22, 23). However, they are not well understood in critically ill neonates due to dynamically changing conditions in the single patient. There is a large inter individual variability in the PK/PD of frequently used medications (antimicrobials, analgosedatives, anti-convulsives, vasopressors, and inotropes) in neonates under critical illness (24) and ECMO (25, 26). These covariates are either predictable (i.e., related to development or drug = maturational determinants), partly predictable (i.e., related to treatment modality), or almost non-predictable (i.e., related to disease = non-maturational covariates). Changes in the Vd and Cl of drugs under critically ill conditions may lead to a high intra- and inter-individual PK variability (for different drugs 30–70%) resulting in either insufficient or toxic plasma concentrations of drugs (27). This may have an impact on the drug disposition and, as a consequence, both under- and over-dosing may contribute to unfavorable outcomes.

### Perinatal Asphyxia—Hypoxia

Perinatal Asphyxia (PA) is defined by the American Academy of Pediatrics (AAP) and the American College of Obstetricians and Gynecologists (ACOG) as a condition of severely deficient supply of oxygen to the body (oxygen deprivation) leading to coma or death (28). In 2009, based on international guidelines, therapeutic hypothermia (HT; 33–34°C) has been recommended to be used for therapy in asphyxiated (moderate to severe) neonates (29). However, the decision to place on ECMO newborns treated for perinatal asphyxia and hypoxic ischemic encephalopathy (HIE) is based on criteria of HIE severity (30). Following perinatal asphyxia, neonates may suffer from HIE (69.4%), respiratory or acute kidney failure (AKI 47–61%), cardiac and hepatic dysfunction, whose rates in the era of HT (31) are similar to the pre-cooling period (32). Multiple organ dysfunction syndrome (MODS), defined as the presence of at least one organ dysfunction in addition to HIE, occurred in 58–88% of asphyxiated neonates (33) and contributed to higher mortality rates (20.5–72.9%) (31). MODS may complicate the course of neonatal ECMO, with a negative impact on survival (34, 35). Moreover, after out of hospital pediatric cardiac arrest, AKI is very common (64% of the enrolled cases, *n* = 282), and severe (41% of the enrolled cases), without difference in incidence in severe AKI between cases that either or not underwent HT (36). As a rule of thumb, *asphyxia* may lead to changes in drug disposition such as decreased or variable drug absorption (AUC, Ka, t_max_ or F), increased (or unchanged) drug distribution (Vd) and decreased drug elimination (CL) (37). However, data on PK changes under asphyxia in neonates are sparse (ceftazidime, amikacin, gentamicin, amoxicillin, and benzylpenicillin) (37–41) and the same holds true for cardiac arrest in neonates and changes in pH (42). Moreover, PK variability in asphyxiated neonates has been reviewed in relation to the impact of HT alone (43–46) or in combination to ECMO (47). Recently, for anticonvulsive drugs such as phenobarbital, which has low hepatic Cl and low protein bound drug HT was not found to be a PK covariate (48, 49), in contrast to birth weight (BW), postnatal age (PNA) (50), and disease severity (51). The disposition of other drugs during neonatal HT has been evaluated in the recent literature (52–56) and the relevant findings are summarized in Table 1.

**Table 1:** Pre-ECMO non-maturational determinants of drug disposition and pharmacology considerations.

**Physiology**
Perfusion status: changes in tissue (regional) perfusion (*↓↑*), organ (systemic) perfusion (*↓↑*), cerebral-, splanchnic-, liver-, and renal flow (*↓↑*), changes in cardiac output (*↓↑*), SVRI (*↓↑*), MODS
Body water status: changes in total water volume (TV), tissue permeability, capillary leakage syndrome, intravascular volume (*↓↑*), extracellular water volume—ECV (*↓↑*)
Acid-base balance: acidosis
Protein status: hypoalbuminemia, α1-acid glycoprotein (increased plasma levels during acute phase decreases free drug plasma levels)
Pharmacology^*^
Absorption (AUC, F, Tmax, Ka): ↓ in asphyxia, ↓ or ↑ or = in sepsis
Distribution (Vd): ↑ or = in asphyxia, ↑ in hydrophilic drugs under sepsis, = in lipophilic drugs under sepsis
Elimination (CL): ↓ in asphyxia, ↓ or ↑ or = based on shock state (hyper/hypo-dynamic) and type of drug elimination (liver or kidney)
Metabolism^*^: ↓ in hypoxia and sepsis
**Physicochemical properties of a drug**
Route of administration: orally administered drugs F = 20–70%, intravenously administered drugs F = 100%
Drug solubility: hydrophilic/ lipophilic drugs, based on octanol/water partition coefficient—LogP - LogP <1 = water soluble - LogP 1–2 = weak water/more lipid soluble - LogP >2 = lipid soluble
Protein binding capacity (albumin, α1-acid glycoprotein): - Low binding <30% - Moderate binding 30–70% - High binding >70%
Elimination via liver: - High hepatic CL drugs = ↑extraction drugs with ↑ intrinsic hepatic metabolizing capacity, dependency on hepatic blood flow - Low hepatic CL drugs = ↓extraction drugs with ↓intrinsic hepatic metabolizing capacity, low dependence on hepatic blood flow, dependency on hepatocellular enzyme activities phase I CYP P 450 and phase II (intracellular oxygen tension, cofactors)
Elimination via kidney: - High/low renal CL drugs, dependency on renal filtration, secretion, and reabsorption
Concomitant medication^*^
Fluid resuscitation: Vd ↑ in hydrophilic drugs under sepsis, no changes in Vd in lipophilic drugs under sepsis
Circulatory support: CL *↓↑* in high/low hepatic lipophilic CL drugs. Renal CL↑ of active metabolites of lipophilic drugs or non-active metabolites of hydrophilic/weak hydrophilic more lipid soluble drugs
Diuretics: renal CL↑ of active metabolites of lipophilic drugs or non-active metabolites of hydrophilic/weak hydrophilic more lipid soluble drugs
Drug-drug PK interactions: Absorption variable or ↓ due to changes in gut perfusion (omeprazole, digoxin, fluconazole). Vd ↓: – competitive protein binding (phenytoin, amiodaron, non-steroidal anti-inflammatory drugs) - Biotransformation *↑↓* for inductors (barbiturates, dexamethasone) or inhibitors (midazolam, fluconazole via CYP 3A4, CYP2A6, CYP2C9, CYP2C19, CYP2D6 a CYP2E1) - Elimination due to filtration *↑↓*, as changes in Vd may lead to changes in CL or changes in perfusion of vas afferent (aminoglycosides, vancomycin) - Tubular secretion *↑↓* (morphine, furosemide) - Reabsorption *↑↓* as a result of drug ionization and urinary pH (benzodiazepines)
Treatment modalities^*^
Therapeutic hypothermia (TH): changes in - Absorption (↓) - Distribution (↓ or ↑, no changes, or variable) - Elimination (↓ or no changes)
Extracorporeal membrane oxygenation (ECMO): changes in - Absorption (↓ or no changes)^*^ - Distribution (↑ or no changes) - Elimination (↑or↓ or no changes)

* *Limited data in neonates*.

*AUC area under the concentration curve, CL clearance, F bioavailability, LogP octanol/water partition coefficient, Ka rate constant of absorption, Tmax—the time at which the Cmax (the maximum serum concentration) is observed, Vd volume of distribution, ↑, increase; ↓, decrease; = no changes*.

### Sepsis/Systemic Inflammatory Response Syndrome

There is lack of consensus for the definition of *sepsis* in neonates (57). So far, the international consensus on pediatric sepsis and SIRS, respectively, was established to address this issue for all children (<18 years old) including term neonates (≥37 weeks completed gestation) in 2005 (58). (59) showed how mortality for MODS, in a pediatric intensive care unit, was significantly higher among term neonates compared with older children (75.4 vs. 50.9%) (59). During sepsis relevant SIRS-related physiological changes occur, which contribute to drug disposition (60). The main physiology and pharmacology considerations in sepsis/septic shock are shown in Table 1. *Sepsis*, and its related factors like tissue (regional) hypoperfusion, MODS (systemic) hypoperfusion, acidosis, hypoalbuminemia, SIRS, type of shock (hyperdynamic/hypodynamic), capillary leakage syndrome, or pharmacotherapy (diuretics, vasopressors, inotropic drugs) may lead to changes in PK, and therefore PD parameters (Cmax, Cmin, AUC0-24/MIC of concentration, and time dependent antibiotics, T > MIC of time-dependent antibiotics, Cmax/MIC of concentration dependent antibiotics). Moreover, sepsis and SIRS may induce a supraphysiologic renal activity, defined as augmented renal clearance (ARC) with enhanced renal pre-load and glomerular hyperfiltration (61). ARC is an established physiological response to hyperdynamic cardiovascular states in adult (61–63) and pediatric critical care patients (64, 65). However, in the neonatal period ARC has not yet been reported. In case of reduced renal functional reserve, secondary to a previously impaired kidney function or worsening organ perfusion, drug clearance may be compromised (64, 66). In children, sepsis has major impact on cytochrome P450 (CYP)3A activity (−90%), as has been illustrated with midazolam as probe drug (67). Such observations should be considered while prescribing drugs for critically ill neonates on ECMO. The interaction between the extracorporeal circuit itself with pre-ECMO disease states needs to be further characterized (68–70).

## The Role of Non-maturational Determinants on Drug Disposition: Focus on ECMO

Extracorporeal membrane oxygenation interferes with the expected attainment of a drug's therapeutic level (71). In the last decades, preclinical and clinical research have provided preliminary evidence of the causative mechanisms for reduced drugs' bioavailability. Pending specific PK studies, the loading dose (LD) is usually based on Vd, while the maintenance dose (MD) is driven by the estimated Cl (72). Moreover, PK changes are strictly dependent on equipment material and circuit design (73). Most data come from *ex vivo* studies on silicone-based oxygenators. Technological advances have added further variability, through the introduction of ever-smaller circuits, new biocompatible coatings and poly-methyl-pentene(PMP) membrane oxygenators (74). Currently, we lack the knowledge of the interaction of contemporary neonatal ECMO circuits and pharmacotherapy. Hereby we summarize the available evidence, stemming from *in vitro* and *in vivo* studies.

### Circuit-Drug Interaction

The modern neonatal circuitry includes cannulas (venous cannula for drainage and arterial cannula for reinfusion or a single double-lumen cannula, when allowed by patients' size), polyvinyl chloride conduit tubing, a centrifugal pump, and PMP hollow-fiber membrane oxygenator (75). Based on patients' conditions, an hemofilter or a continuous renal replacement therapy may be added to the circuit design (75).

Both size and material of each of the above-mentioned components may lead to significant PK changes as a result of three main mechanisms: (i) sequestration into the circuit; (ii) increased Vd; and (iii) altered Cl.

### Drugs' Sequestration by the ECMO Circuit: Components and Materials

Significant extraction of medications occurs in off-patient ECMO systems as a result of a complex interaction among circuit components and specific physiochemical properties of drugs, notably molecular weight, ionization, hydrophilicity, and protein binding (13, 15). The octanol-water partition coefficient (LogP) is a measure of a drug's lipophilicity (76). The higher the LogP (>2), the higher the drug sequestration (13, 15, 77). Similarly, highly protein bound drugs are more prone to be adsorbed into the ECMO systems (14). These *ex vivo* findings were confirmed by *in vivo* ovine ECMO models (77).

Equipment matters, as different materials of oxygenators, tubing, coating, and pumps may have a variable impact on drug disposition (15, 78–81).

Pediatric membrane oxygenator technology underwent significant advancements over the last decades (82). Improvement of materials, surface area and priming volume may support pharmacotherapy. Indeed, the variability of drug adsorption by different membrane oxygenators has been acknowledged since the early 90's (71, 83). Lipophilic drugs were largely sequestered into silicone membranes, at variance with the polypropylene ones (15, 71, 83). Similarly, the last-generation polymethylpentene hollow fiber oxygenators have shown less drug adsorption when compared to silicone-based membranes, especially for the lipophilic drugs in the first hours after injection in off-patients experiments (84).

Polyvinylchloride (PVC) tubing was found to be the primary site for drug sequestration (85). According to *in vitro* data, fentanyl was lost to the PVC tubing by 80% after 120 min, with an additional 5% lost to the oxygenators (85). Polymethylpentene-based oxygenator had a slightly higher impact on fentanyl disposition, if compared to the microporous polypropylene-based one (85). In the same study, morphine was lost to the PVC tubing by 40% after 5 min, with almost no further adsorption by the oxygenators (85). In contrast, recombinant human albumin/heparin coating tubing showed no effect on disposition of hydrophilic drugs, such as cephalosporine and carbapenems (86). These findings were further supported in a more recent *ex vivo* study, which evaluated beta-lactams in ECMO circuits made up of polymethylpentene membrane, centrifugal pump, heat exchanger, and PVC tubing (87). Results confirmed that beta-lactams (except for ceftriaxone) were not sequestered into the circuit (87).

Although the impact of coating has been neglected for years, more recent *in vitro* studies provided evidence that surface modification may affect drug disposition to some extent (88). Coating is meant to mimic the endothelial surface to enhance biocompatibility and it is generally defined as bioactive, when it is based on heparin and nitric oxide, or biopassive, if albumin and polymers such as phosphorylcholine are used (89). *In vitro* results from a study specifically designed to investigate the influence of coating on morphine and fentanyl disposition have shown that the following four types of coating were inert to drug absorption: synthetic albumin, heparin-free biopassive polymer, recombinant human albumin ± heparin, and covalently bonded heparin coatings. In contrast, two other types of surface modifications were associated at 5 min with a significant reduction of morphine levels: poly2methoxylacrylate polymer and covalently bonded heparin (88). No significant differences were reported for fentanyl concentrations (88). These findings further illustrate how drug disposition results from a complex chemical and molecular interaction between individual drugs and ECMO components' individual characteristics. Indeed, electrochemical properties, namely the electric charge and degree of hydrophilicity of surface coatings, may contribute to modulate drugs' sequestration (90).

Although the influence of the type of pump itself has not been defined, centrifugal pump-based circuits with hollow-fiber membrane oxygenators have shown the least absorption for all drugs (13, 15), and this phenomenon is most pronounced for lipophilic drugs (15). Besides chemical drivers, mechanics could be advocated to affect drugs' PK, as blood is constantly exposed to variable pressures and flow-rates over the extracorporeal run (79). ECMO blood-flow is thought to affect drugs' PK (91), nevertheless the specific impact of blood-flow variability has not yet been characterized.

In addition, the type of priming solution and temperature are involved in the complex chemical mechanisms of drug loss and stability during ECMO (92), as explained in the next paragraph. Circuit age further affects pharmacotherapy: on one hand the saturation of binding sites may smooth the tubing impact on PK; on the other hand, it is not clear if the circuit acts as a reservoir, by releasing drugs back into the patient with a potential risk of cumulative effect and late toxicity (13, 71, 93). Thus far, the *in vitro* circuit-drug interaction has been characterized over 24 h, no data are available beyond this time frame.

### ECMO-Induced Volume of Distribution Increase

The connection of a neonate to the extracorporeal circuit will affect the apparent Vd of drugs, through three main mechanisms. Firstly, as previously mentioned, the direct drug adsorption into the circuit is the driving factor (71, 93). Secondly, the haemodilution from the priming solution has been advocated for ECMO-related PK variability (92). In neonates, the priming volume of contemporary circuits approximates 250–300 ml, which equals the circulatory volume of a 3 kg neonate. Furthermore, over the course of an ECMO run, the frequent administration of blood products and crystalloids contribute to worsen the hemodilution (94). Hydrophilic drugs are the most affected, as their Vd is limited to the extracellular compartment, with no intracellular drug reservoir available for retrograde diffusion (72). The extension of the plasma compartment during the ECMO start or in critical illness affects the LD, which is the first dose needed to guarantee the therapeutic concentration (72, 95). LD is directly proportional to the enlarged Vd and, hence, should be increased accordingly (95). The priming dilution, in conjunction with electrolytes and temperature perturbations, may affect also plasma proteins, especially albumin and alpha_1_-acid glycoprotein, thus altering the plasmatic drug-binding (14). Hypoalbuminemia is a multifactorial process, which results from ECMO- and disease-driven physio-pathologic changes (96). The increase of unbound or free drugs may expose ECMO neonates to potential toxicity (97). Lastly, the ECMO-related physiologic changes and the underlying disease state influence Vd, as a result of the systemic inflammatory response (98–102).

### ECMO-Induced Clearance Variability

Drug clearance relies on kidney and liver function, which are usually altered on ECMO, as a result of the clinical status and circuit-related factors (73, 94, 103). In the early phase of extracorporeal circulation, the SIRS releases inflammatory mediators and endogenous cytokines, thus leading to vasodilatation, increased cardiac output and renal perfusion (73, 94). In veno-arterial ECMO, non-pulsatile blood flow is associated with a reduction of the glomerular filtration rate (104). Moreover, the inflammatory state of the critically-ill is associated with the downregulation of the expression and activity of cytochrome P450 enzymes involved in the hepatic drug metabolism (67). Low clearance and consequent rise of drug levels might expose the patient to increased pharmacological effect and toxicity (67, 73).

## Disposition of Analgo-Sedatives on Neonatal ECMO

During ECMO, neonates are exposed to multiple sedatives and analgesics, mostly for prolonged periods, to provide comfort, pain relief, and safety (105). The extracorporeal circuit has a large impact on sedatives and analgesics disposition, leading to high sedative needs (106–109). Drug physicochemical properties may assist in the dose prediction, which is titrated to clinical effect (5). Indeed, lipophilic agents, like fentanyl, propofol, and midazolam are highly sequestered into the circuit (15, 93, 110), especially in the first hours of bypass (84).

In the neonatal age, prolonged and sustained analgo-sedation is associated with clinical relevant adverse effects such as tolerance, dependency, impaired brain development, and iatrogenic withdrawal syndrome (105, 111). Among opioid-sparing strategies, the daily interruption of sedation and analgesia was shown to be feasible, safe, and effective (112). However, sedation targets differ among ECMO centers, ranging from deep to conscious sedation practice (5, 113). The use of alternative non-opioid agents should be preferred (73, 84). Morphine and paracetamol have a favorable PK profile (15, 84, 114), while preliminary data on α_2_-adrenergic agonists dexmedetomidine and clonidine suggest the need for increased dosing (81, 115).

In this section we will summarize current evidence of the disposition of sedatives and analgesics on contemporary neonatal ECMO circuits (Tables 2, 3) (15, 83, 84, 91, 93, 108, 110, 114, 115, 117–120, 134).

**Table 2:** *In vitro* PK datasets of contemporary neonatal ECMO circuits.

**References**	**Drug**	**Pump**	**Timing (h)**	**Drug loss (%)**
Wildschut et al. (15)	Cefotaxime	Centrifugal	3	2
Cies et al. (116)	Daptomycin	Centrifugal	24	0
Wagner et al. (81)	Dexmedetomidine	Roller	24	76–90
Nasr et al. (110)	Dexmedetomidine	Centrifugal	48	51
Wildschut et al. (15)	Fentanyl	Centrifugal	3	66
Raffaeli et al. (84)	Fentanyl	Centrifugal	24	84
Nasr et al. (110)	Fentanyl	Centrifugal	48	68
Wildschut et al. (15)	Meropenem	Centrifugal	3	11
Wildschut et al. (15)	Midazolam	Centrifugal	3	36
Raffaeli et al. (84)	Midazolam	Centrifugal	24	40
Nasr et al. (110)	Midazolam	Centrifugal	48	26
Wildschut et al. (15)	Morphine	Centrifugal	3	68
Raffaeli et al. (84)	Morphine	Centrifugal	24	51
Nasr et al. (110)	Morphine	Centrifugal	48	4
Wildschut et al. (15)	Paracetamol	Centrifugal	3	56
Gillogly et al. (114)	Paracetamol	Roller	6	0
Raffaeli et al. (84)	Paracetamol	Centrifugal	24	49
Raffaeli et al. (84)	Sufentanil	Centrifugal	24	83
Wildschut et al. (15)	Vancomycin	Centrifugal	3	33

*All experiments were reported to be performed on new and blood-primed circuits, based on hollow-fiber membrane oxygenators. Sample site is pre-membrane*.

**Table 3:** Summary of drug physicochemical properties, ECMO-induced PK changes, and drug dosing.

**Drug class**	**Medication**	**LogP**	**PB%**	**ECMO-related PK changes**	**Standard dosing in critically ill term neonates**	**Dosing recommendation for neonates on ECMO**	**References**
Benzodiazepine	Midazolam	3.89	97	*In vitro*: moderate sequestration Clinical PK: Increased Vd	Loading dose 50–150 mcg/kg Maintenance dose 10–60 mcg/kg/h	Consider increasing the loading dose in the early phase of ECMO. Beware of drug/metabolites accumulation over time.	(15, 84, 93, 108, 109, 117, 118)
α_2_-adrenergic agonist	Dexmedetomidine	3.39	94	*In vitro*: moderate sequestration	Loading dose 1 mcg/kg	Although sparse, data suggest the need for a loading dose.	(81, 110, 118)
				No clinical PK	Maintenance dose 0.2–0.7 mcg/kg/h		
	Clonidine	1.59	20–40	Clinical PK: increased Vd and clearance in a population PK study of ECMO with CVVH	Maintenance dose 0.1–1 mcg/kg/h	Although sparse, data suggest the need for higher doses.	(115, 118)
Opioid analgesics	Morphine	0.99	30–40	*In vitro*: mild to moderate drug loss in contemporary ECMO systems	Loading dose 100 mcg/kg	Analgesic of choice during ECMO at most centers. Minimal dose adjustment may be required.	(15, 84, 91, 118, 119)
				Clinical PK studies (older circuits): no changes	Maintenance dose 10–40 mcg/kg/h		
	Fentanyl	4.12	80–85	*In vitro*: high drug loss	Loading dose 0.5–3 mcg/kg	Consider alternative drugs. Consider increasing the dose, when used for procedural analgesia	(15, 83, 84, 93, 118)
				Clinical PK: need for higher doses	Maintenance dose 0.5–2 mcg/kg/h		
	Sufentanil	3.4	NA	*In vitro*: high drug loss	(Pediatric dosage) Loading dose 0.25–2 mcg/kg	Limited data for dosing recommendations.	(84, 118, 120)
				No clinical PK available	Maintenance dose 0.5–1.5 mcg/kg/h		
Anesthetic (phenolic derivative)	Propofol	3.79	95–99	*In vitro*: high drug lossNo clinical PK available	Bolus 2.5 mg/kg	Drug-related toxicity, propofol-related infusion syndrome PRIS call for caution in the use of propofol during neonatal ECMO	(93, 118)
Non-opioid analgesics	Paracetamol	0.51	25	*In vitro*: conflicting data	7.5 mg/kg/6h	Limited data for dosing recommendations.	(15, 84, 114, 118)
				No clinical PK.			
b-Lactam	Ampicillin	1.35	15–30	*In vitro*: increased Vd, low-moderate drug sequestration	50–70 mg/kg/8 h	Standard dosing, given the large therapeutic window.	(92, 118)
				No clinical PK			
	Cefotaxime	−1.4	35	*In vitro*: low drug sequestration in contemporary systems	Postnatal age <7 days: 100–150 mg/kg/day in 2 or 3 doses	Standard dosing; perform TDM to verify adequate supra-MIC levels.	(15, 118, 121)
				Clinical PK: standard dosing is effective	Postnatal age 7–28 days: 150–200 mg/kg/day in 3 or 4 doses		
	Meropenem	−0.69	2	*In vitro*: large increase in Vd and low-moderate drug loss	Postnatal age <7 days: 20 mg/kg every 12 h	Standard dosing. Perform TDM to verify adequate supra-MIC levels. Consider higher dosing or continuous infusion in case of increased clearance or RRT.	(15, 118, 122)
				Clinical PK: increased Vd and increased Cl, in ECLS + RRT	Postnatal age 7–28 days:20 mg/kg every 8 h Meningitis: 40 mg/kg every 8 h		
Glycopeptide	Vancomycin	−1.4	50	*In vitro*: large increase in Vd, minimal to moderate loss Conflicting clinical PK data in contemporary circuits, in terms of impact on Cl	Postnatal age <7 days: 10–15 mg/kg every 8/12 h >7 days: 15 mg/kg every 6/8 h	Dosing guidelines based on age and renal clearance. Suggested dose in neonates: 25 mg/kg/dose every 12–24 h. TDM for dosing monitoring and adjustement.	(3, 15, 92, 118, 123–125)
Aminoglycoside	Gentamicin	−3.1	0–30	*In vitro*: increased Vd, reduced Cl. Low drug sequestration	Term neonates with normal renal function: 3.5–5 mg/kg every 24 h	Dosing guidelines based on age and renal clearance. Initial dose 2.5 mg/kg/dose every 18 h, subsequent doses individualized through TDM. Once ECMO is discontinued, dosage should be readjusted according to body water shifts.	(3, 118, 126–129)
Antiviral	Oseltamivir	1.3	3	NA	3 mg/kg/dose every 12 h, orally	Standard dosing	(118, 130, 131)
Azole anti-fungal	Fluconazole	0.5	11–12	*In vitro*: minimal sequestration PK-PD study available	Prophylaxis: 3 mg/kg every 72 h	Loading dose 12 mg/kg followed by 6 mg/kg/die	(118, 132, 133)

*CVVH, continuous veno-venous hemofiltration; Vd, volume of distribution; NA, not available; PK, pharmacokinetics; PRIS, propofol-related infusion syndrome; RRT, renal replacement therapy; TDM, therapeutic drug monitoring*.

### Benzodiazepines

Midazolam has been extensively studied in the neonatal ECMO population. Moderate sequestration into the circuit has been observed through *in vitro* experiments, based on both old (15, 93) and contemporary circuits (15, 84). Two PK studies are available in the neonatal ECMO population, with contrasting results. Although both described the increase of Vd, since the start of ECMO (108, 117), Mulla et al. found a constant Cl of midazolam in neonates on veno-venous ECMO, with a prolonged elimination half-life leading to drug accumulation after 48 h (108). In contrast, Ahsman et al. reported the increase of midazolam Cl over time in neonates on veno-arterial ECMO (117). These PK data suggest the need for an increased LD in the early phase (first 24–48 h) of extracorporeal support, following which dosage should be titrated down, given the risk of accumulation of midazolam and its metabolites (108, 117) and, consequently, prolonged sedation (135).

### Opioids

Fentanyl is highly sequestered into the circuit (15, 92) and dose escalation is required in neonates and infants exposed to extracorporeal circuits (107, 136). Despite the technological improvements, the impact of contemporary hollow-fiber-based oxygenators remains high for lipophilic drugs, such as fentanyl and sufentanil (84). Most centers use morphine as analgesic and sedative during neonatal ECMO, because its PK profile is not significantly altered. Clinical PK studies have reported a two-fold increase of morphine Vd (91). The Cl decreased following ECMO cannulation (119, 134) but increased over time, in relation to creatinine clearance, reflecting age-related maturation of drug excretion (91). Moreover, when compared to fentanyl, morphine continuous infusions were associated with improved analgesia, reduced drug withdrawal and length of stay (137). Therefore, morphine remains the opioid of choice for neonatal ECMO. Dose adjustments need to be titrated to clinical sedo-analgesic targets, pending evidence on contemporary circuitry-related PK.

### Non-opioid Analgesics

Based on preliminary *in vitro* studies, paracetamol has been suggested as a promising analgesic during neonatal ECMO (84, 114). However, clinical PK evaluations are needed to provide dosing recommendations.

### Propofol

This highly lipophilic and protein-bound sedative-hypnotic agent is largely sequestered into the ECMO circuit (93). The drug-related toxicity and concerns for propofol infusion syndrome (PRIS) (138) call for caution in the prolonged use of this drug during neonatal ECMO.

### α_2_-Adrenergic Agonists

Clonidine use and prescription during neonatal and pediatric ECMO is supported by a recent population PK study, which suggested higher clonidine doses, based on the increase of Vd and Cl in the specific setting of ECMO and renal replacement therapy (115). Limited *in vitro* data are available for dexmedetomidine, which is partially sequestered into the circuit: a LD may be required, although recommendations for its long-term use cannot be provided (81).

## Disposition of Antimicrobial and Antiviral Drugs During Neonatal ECMO

**Figure 1:**
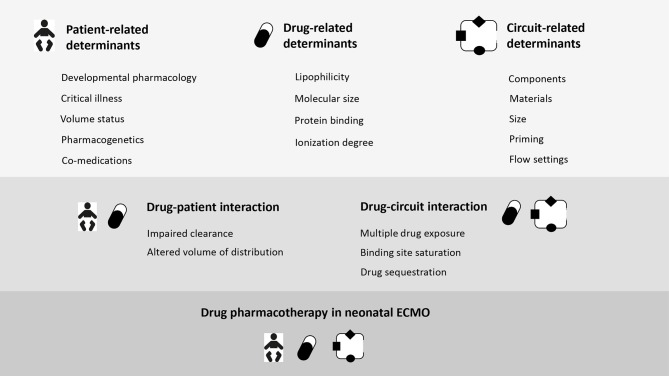
Determinants of drug disposition during neonatal ECMO.

Infection remains a real threat for critically-ill neonates on ECMO, with an incidence rate of 5.4 and 5.7% in respiratory and cardiac runs, and reduced survival to 51 and 19%, respectively (34). A timely and adequate antimicrobial therapy is therefore pivotal to improve outcomes (139). However, the goal to provide optimal antibiotic therapy is impaired by the ECMO-induced PK changes, which can be only partially predicted, based on current knowledge on drug-circuit-patient interaction (Figure 1) (13, 15, 103). Moreover, antimicrobial prescribing is further complicated by the lack of clinical titratable endpoints (103). Therefore, PK and PD remain the best available predictors of antimicrobial efficacy. Pending evidence-based pharmacotherapy guidelines, neonates on ECMO are still at risk of sub-optimal antibiotic exposure, contributing to treatment failure and bacterial resistance (26). In this section we will summarize current evidence of antimicrobial bioavailability on contemporary neonatal ECMO circuits (Tables 2–4) (4, 15, 92, 116, 118, 121–133).

**Table 4:** Overview of the Pubmed (ecmo, newborn, pharmacokinetics, *n* = 72) search on pharmacokinetics of 16 different compounds.

**References**	**Compound**	Protein binding^*^	pKa^*^	**Comments**
Pokorna et al. (140)	Phenobarbital	20–45%	8.14 (acid)	Hepatic, mostly via CYP2C19
Cies et al. (123)	Vancomycin	50%	2.99 (acid)	Mainly by renal route, renal transporters likely involved
			9.93 (basic)	
Kleiber et al. (115)	Clonidine	20–40%	8.16 (basic)	Renal (about 50%) and hepatic (about 50%), including CYP2D6
Niimi et al. (141)	Anti-thrombin	n.a.	n.a.	Protein, no specific elimination routes described
Watt et al. (132)	Fluconazole	11–12%	12.71 (acid)	Renal (90%) and hepatic (10%)
			2.56 (basic)	
Ahsman et al. (117)	Midazolam	97%	6.57 (basic)	Intestinal and hepatic metabolism, CYP3A
Ahsman et al. (121)	Cefotaxime	n.a.	3.18 (acid)	Renal elimination (20–40%) and metabolism (desacetyl derivative is the major metabolite)
			4.15 (basic)	
Ahsman et al. (142)	Sildenafil	96%	7.29 (acid)	Hepatic metabolism, CYP3A4 > 2C9
			5.97 (basic)	
Kendrick et al. (143)	Amiodarone	>96%	8.47 (basic)	Almost exclusively hepatic, CYP2B8
Peters et al. (144)	Morphine	30–40%	10.92 (acid)	90% hepatic, glucuronidation >> demethylation
			9.12 (basic)	
Mulla et al. (145)	Theophylline	40%	7.82 (acid)	Hepatic metabolism, demethylation, hydroxylation and N-methylation (CYP1A2) to caffeine
			−0.78 (basic)	
Wells et al. (146)	Ranitidine	15%	8.08 (basic)	N-oxidation is the most relevant metabolite
Aebi et al. (147)	Ribavirin	n.a.	11.88 (acid)	(de)phosphorylation
			−1.2 (basic)	
Wells et al. (148)	Bumetanide	97%	4.69 (acid)	45% primary renal, oxidation 55%
			2.7 (basic)	
Bhatt-Metha et al. (129)	Gentamicin	Low, 0–30%	12.55 (acid)	Primary renal, by glomerular filtration
			10.18 (basic)	
Pokorna et al. (149)	Sufentanil	79–93% (alpha-acid glycoprotein)	8.86 (basic)	Hepatic, oxidative N-, and O-dealkylation

* *Data on protein binding and pKa values were retrieved on www.drugbank.ca [CYP, cytochrome P450; n.a., not available/applicable]*.

### Beta-Lactams

Beta-lactams are hydrophilic time-dependent antimicrobials, with a variable degree of protein binding and renal elimination (150). Their killing activity is strictly related to the time the unbound drug is above the minimum inhibitory concentration (MIC). The knowledge of ECMO-related PK changes is limited in novel circuitry and further complicated by the well-known instability (i.e., temperature) of this class of antibiotics (151). Based on *in vitro* observations, ampicillin showed a moderate loss in older silicon-based neonatal systems (92). The impact of contemporary circuits on cefotaxime seems negligible (15).

Although the Vd of cefotaxime was increased during ECMO, Cl was comparable to the one of non-ECMO neonates (121). Based on a neonatal PK study, standard dosing regimen of cefotaxime during ECMO provided supra-MIC plasma levels (121). Therefore, given the large therapeutic window of cefotaxime, dose adjustments are usually not needed.

Broad-spectrum carbapenem agents, such as meropenem, may be required over the neonatal ECMO course (139). The impact of the ECMO circuit on drug disposition consists of a moderate drug sequestration (15), larger Vd, and higher clearance (152). The latter factor is magnified when renal replacement therapy is added to the circuit design (122).

### Glycopeptides

Vancomycin is a hydrophilic time-dependent antimicrobial, largely used in the NICUs for treatment of Gram-positive infections (118, 153). Given the narrow therapeutic window and the risk of nephrotoxicity, the PK profile of vancomycin has been extensively evaluated both *in vitro* and *in vivo* neonatal settings since the 90's (154–156). Vancomycin Cl is strictly related to renal function (155, 157) and the drug half-life was found to be prolonged in ECMO patients (156). However, these findings referred to older roller pump-based systems. Although data on contemporary circuits are limited, recent neonatal PK studies have revealed enhanced Cl, potentially leading to under-exposure (123). An empiric dosing strategy of 25–30 mg/kg/dose every 12–24 h is suggested, with a close therapeutic drug monitoring (TDM) (125).

Continuous vancomycin infusions were found to be associated with earlier and improved attainment of target concentrations compared to the intermittent modality in neonates, with no difference in terms of adverse effects (158). However, no evidence is available for the optimal infusion modality during ECMO.

Another glycopeptide antimicrobial which may be used during neonatal ECMO is teicoplanin. Although specific neonatal data of teicoplanin disposition in the extracorporeal setting are lacking, the evidence from an adult PK study suggests the need for higher doses during ECMO (159). In this prospective population PK evaluation, the predictive target attainment was reduced during ECMO for every simulated dosing, despite the Vd was lower and Cl was not affected by the extracorporeal circuit (159). Based on the hydrophilic profile of the drug, the hemodilution and protein binding could be addressed as the main drivers for teicoplanin disposition on ECMO (159).

### Aminoglycosides

Gentamicin is a hydrophilic antimicrobial with a relatively low protein binding, largely used in the NICUs for the treatment of infections due to Gram-negative bacteria (118, 153). During ECMO, gentamicin has been found to have an increased Vd, as a result of the large exogenous blood volume for circuit priming and decreased Cl, leading to a prolonged elimination half-life (4, 126, 128). The renal dysfunction, which is a common multifactorial condition during ECMO, may be considered as the main determinant of the prolonged elimination half-life of gentamicin (72). Given the concentration-dependent antimicrobial activity of aminoglycosides, it is highly recommended to perform TDM to ensure adequate antimicrobial exposure.

### Antivirals

Oseltamivir is a neuraminidase inhibitor of both type A and B influenza virus (160). This drug is approved by the Food and Drug Administration (FDA) for the treatment of children older than 2 weeks of age with flu (130, 161). Oseltamivir is an oral pro-drug which is rapidly converted to oseltamivir carboxylate, the active metabolite (150, 160). Based on previous pediatric PK ECMO case series, the impact of ECMO on oseltamivir disposition is negligible with no need for dosing adjustment (131). However, oral bioavailability was reported to decrease in patients with impaired gastric motility and enteral absorption (131). Although evidence in the neonatal setting is scant, adult data support the lack of effect of ECMO on the oseltamivir's PK (162, 163).

## From Fragmented Data to Integrated Knowledge

Obviously, also in neonates and children on ECMO, pharmacotherapy is a very important tool in the medical management. As a result of the large PK-PD variability, drug dosing is only to a very limited extent validated in the setting of neonatal ECMO (164). Methodological development within the field of clinical pharmacology and modeling should assist ECMO physicians to improve our practices. The other way around, modelers will need the data to get this job done.

A knowledge-driven improvement strategy necessitates sufficient understanding of human developmental biology to subsequently translate such knowledge into prediction differences in drug absorption, distribution, metabolism, and excretion (PK). Only once this PK is sufficiently well covered, an appreciation of the developmental aspects of drug-receptor or -target interactions (PD) can be considered. Physiologically-based PK (PBPK) modeling is such a structured approach to translate knowledge into prediction, but the development of such modeling techniques necessitates the collaboration of clinicians with researchers specifically skilled in modeling techniques (165). PBPK approaches provide a potent systematic way to make the most of already acquired knowledge (physiology, system knowledge) to adapt drug dosing to the needs of children on ECMO, as has recently been illustrated for fluconazole (166).

The aim is not to describe the workflow and technical details related to the development of pediatric or neonatal PBPK model (167, 168), but to illustrate how ECMO physicians and clinical researchers can contribute to improved ECMO-related pharmacotherapy in neonates and children by generating data on ECMO related (patho)-physiology, including aspects related to the initial indication to initiate ECMO, and by sharing PK datasets and data on neonatal and pediatric equipment.

### PBPK Methodology

**Figure 2:**
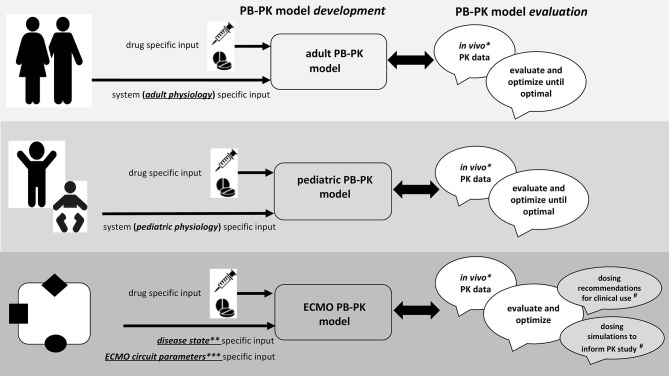
Integrated approach for drug prediction through physiologically-based pharmacokinetic (PBPK) models during neonatal ECMO.

In essence, PBPK is a structured method for data integration, hypothesis testing and knowledge generation (167, 168). Moreover, one may check consistency of data obtained from different sources (*in vitro, in vivo, in silico*) or predict outcome (PK, PD) of future experiments, hereby enabling decision making or optimization of study design. PBPK (“so-called bottom-up”) applies mathematical models for mechanistic integration of pharmacology principles, assumptions, and data along the drug development process. It hereby integrates different types of information, such as clinical data and *in silico, in vitro*, and *in vivo* observations. PBPK hereby explicitly discriminates between physiological properties of the population (system parameters, like cardiac output, renal function, liver size, weight, plasma protein, different between populations) and compound specific (chemical, pH, solubility) properties, not different between populations (Figure 2). Using this approach, it has applications in drug development for first-in-human, first-in-child, or first in ECMO-patients, and became an established tool for drug development and regulatory needs, like e.g., data in cases with hepatic or renal impairment, drug-drug or drug-food interactions to avoid the need to recruit an impossible number of patients with very specific issues while still have sufficient confidence in the dosing regimens. The final intention is to generate dosing recommendations, or alternatively, simulations to subsequently conduct PK studies, as highlighted in Figure 2 for the specific ECMO setting (^**#**^).

### Why ECMO Physicians and Clinical Researchers Are Needed to Develop Such Models

As illustrated in Figure 2, the workflow to develop and build confidence in PBPK models tailored to neonatal and pediatric ECMO pharmacotherapy necessitates availability of *in vivo* PK data (^*^), data on disease state (^**^), and on ECMO circuit parameters (^***^), and this is exactly why clinicians should become aware of the usefulness of such data beyond compound specific relevance (164, 165, 167, 168).

#### Availability of *in vivo* PK Dataset (^*^, Figure 2)

To illustrate that there are indeed already quite some compound specific PK observations, we conducted a structured search in PubMed on 15 January 2019 with [*ECMO, newborn*, and *pharmacokinetics*] as search terms. This resulted in 72 hits, and additional search with “infant” resulted in 79 hits, but no additional compounds. The results of this search are provided in Table 4, reporting on the compounds (*n* = 16) retrieved and supported by the most recent reference (115, 117, 121, 123, 132, 140–149). From a PBPK perspective, it is important to realize that these compounds are quite different when we consider protein binding, pKa (reflecting the chemical characteristics of the compound) and can be used to evaluate and optimize a variety of elimination routes, including renal, phase 1, and phase 2 processes: a perfect mix to validate the models. So PK data sharing and collaboration is an obvious need, since the data already exist and can be used to predict PK for drugs not yet evaluated or even not yet marketed (169).

#### Data on Disease State (^**^, Figure 2)

Extracorporeal membrane oxygenation is a technique to treat life threatening conditions, so data on these underlying disease conditions are also needed to further develop ECMO related P‘BPK models: *it's is not just the technique, but also the reason for the technique that matters*. To illustrate the feasibility to integrated (patho) physiology, we refer to PBPK models for carvedilol in children with cardiac failure (170) or intensive care adult patients with hypo-albuminemia (171). In the neonatal and pediatric ECMO, perinatal asphyxia, sepsis, or post resuscitation are common settings. It has been proven that disease affects drug PK (*refer to previous section on pre-ECMO disease*) (37, 67). Besides such observations, clinical researchers should also consider to build multi-center datasets (as part of the ongoing ELSO registry initiatives) on inter- and intra-patient trends of “real world” data. We hereby refer to trends in fluid retention, albumin, creatinine, heart rate, and cardiac output, energy expenditure or more specific issues like alfa-1 glycoprotein (Table 4). This is because such datasets can further feed and improve PBPK prediction, including intra-patient trends with time (8, 172). This has also recently been illustrated for e.g., alfa-1 glycoprotein maturation (173).

#### Data on ECMO Circuit Parameters (^***^)

Finally, equipment matters and data on newer extracorporeal technology need to be considered. PBPK modeling will generate further knowledge, which may guide both the development of new ECMO devices and the refinement of current technology at a biomedical engineering level.

## Conclusions and Future Directions

Extracorporeal membrane oxygenation has an established role in the care of critically ill neonates. The exposure to the extracorporeal circuit impacts on drugs' disposition, potentially leading to undertreatment or toxicity, especially for drugs with a narrow therapeutic index. Non-maturational determinants (such as asphyxia/hypoxia, sepsis/SIRS, MODS) during pre-ECMO predetermine large Vd for hydrophilic drugs due to the underlying disease, while superimposed ECMO may lead to larger Vd for lipophilic and, to a lesser extent, hydrophilic drugs. Therefore, LD adjustment may be recommended to achieve optimal drug levels in neonates on ECMO. CL is influenced by renal (hydrophilic, high renal clearance drugs) and/or hepatic functions (lipophilic, high liver clearance drugs) under sepsis, asphyxia and treatment modalities (HT, ECMO), and optimal maintenance dose adjustment should be achieved on an individual basis (development, disease, genetics). Therefore, TDM is suggested to optimize LD/MD in these critically ill neonates. As drug dosing needs to be guided by PK or PD or PK/PD principles, the understanding of PK-PD changes during (pre-) ECMO will assist in the prescribing optimization and, eventually, contribute to improve patients' outcomes.

In this review we have provided an overview of the available evidence on the impact of both maturational and non-maturational determinants of PK in critically-ill neonates on ECMO. We subsequently have discussed the relevance of these determinants on the disposition of analgo-sedatives and antimicrobial and antiviral drugs during neonatal ECMO. Future efforts should be directed toward a more integrated approach, by combining existing knowledge to predict PK profile. *Sparse samplings* of three different periods (pre-, during, post- ECMO) may be adopted to better understand dynamically changing drug disposition. Further *PK in vivo*/*in vitro studies* will provide insights into the role of contemporary ECMO systems superimposed on maturational/non-maturational determinants.

Gathered knowledge into the maturational physiology-, illness-, and ECMO-related PK impact should be used to inform *PBPK modeling*, which is emerging as an alternative and powerful tool to provide bedside dosing guidance. Lastly, *a prospective validation of PK/PD studies* is needed by well conducted clinical trials to optimize dosing.

The final aim will be to apply pharmacotherapy in a goal-directed fashion, by reaching optimal PD outcomes through the *individualization* of the prescription, thus maximizing the therapeutical benefits in these vulnerable patients.

## Author's Note

PP and DT represent the section of Pharmacology of the European Society of Pediatric and Neonatal Intensive Care (ESPNIC).

## Author Contributions

All authors contributed conception and design of the article. GR wrote the first draft of the manuscript. PP, KA, and DT wrote sections of the manuscript. All authors contributed to manuscript critical revision, read and approved the submitted version.

### Conflict of Interest Statement

The authors declare that the research was conducted in the absence of any commercial or financial relationships that could be construed as a potential conflict of interest.
